# Acetabular osteometric standards for sex estimation in contemporary Croatian population

**DOI:** 10.3325/cmj.2019.60.221

**Published:** 2019-06

**Authors:** Pero Bubalo, Marija Baković, Martina Tkalčić, Vedrana Petrovečki, Davor Mayer

**Affiliations:** Institute of Forensic Medicine and Criminalistics, University of Zagreb School of Medicine, Zagreb, Croatia

## Abstract

**Aim:**

To determine the sexual dimorphism of acetabular measurements in contemporary Croatian population and to provide a discriminant function equation for sex estimation.

**Methods:**

The sample consisted of 200 adult pelvic bones (100 male and 100 female) from positively identified victims of Croatian War of Independence. In total, 96 left (48 male and 48 female) and 104 right (52 male and 52 female) acetabula were measured. One author measured two acetabular parameters using a sliding caliper: acetabular diameter (AD) and transverse acetabular diameter (TAD). Another author re-measured the acetabula of 40 randomly selected individuals to determine the inter-observer error.

**Results:**

Both measured variables showed significant sexual dimorphism. Men had significantly higher values for AD and TAD than women. Receiver operating characteristic curve analysis showed that the cut-off point for prediction of male sex when using acetabular diameter was higher than 54 mm. For transverse acetabular diameter it was higher than 52 mm. The discriminant function was generated by using both acetabular variables, with 88% of accuracy in sex estimation. Inter-observer error was not significant.

**Conclusion:**

The acetabular measurements can be used for sex estimation in contemporary Croatian population with high accuracy.

Sex estimation of human skeletal remains is of crucial importance in medicolegal context, as it is usually the first step in the identification of remains (1-3). Two methods of sex estimation are commonly used – morphological and osteometric (4,5). As the morphological method is based on the visual evaluation of sexually dimorphic morphological bone features, it is prone to subjective judgment and depends on the examiner’s experience. The need to find more reliable and objective methods led to the development of osteometric methods. These are based on statistical analyses of measurements of different skeleton parts, meaning that formulae are generated from discriminant function analysis (6-10). One of the main problems of osteometric method is inter-observer difference. Namely, it is hard to precisely define the starting and finishing point of each measurement, which leads to different readings of the same measurement by two observers (11). In addition, bone fragmentation and damage resulting from body decay decreases the number of measurements that can be obtained. Another problem is that formulae generated from discriminant function analysis are population-specific, as various populations differ with regard to general body size and degree of sexual dimorphism, and are not as reliable outside the population they were based on (6,7,12,13). However, some recent studies indicate that some formulae are possibly less population-specific (14,15).

The aim of this study was to develop standards for sex estimation of Croatian population by using two acetabular parameters. The acetabulum was chosen as one of the most diagnostic pelvic variables for sex assessment and one of the body parts that are less vulnerable to post mortem decay and damage. To our knowledge this is the first study in Croatia and the surrounding region to use pelvic bone parameters in developing population-specific standards for sex assessment.

## Materials and methods

The study sample were the skeletal remains of the contemporary Croatian population, precisely, the victims of Croatian War of Independence (1991-1995). The sample consisted of pelvic bones from 100 male and 100 female victims who had already been positively identified.

Victims had been identified at our Institute using various methods, including anthropological examination, dental records, antemortem x-ray, and DNA analysis. The sample includes individuals from all socioeconomic categories and Croatian regions, with the mean age of 51 years (mean age for men 45.3 years, range 20-76; mean age for women 57.1 years, range 34-80). Only complete, undamaged acetabula were measured. If both acetabula were complete, only the better preserved one was measured. Consequently, the measurements were performed on 96 left (48 male and 48 female) and 104 right acetabula (52 male and 52 female). All of the measurements were made in period from 2014 to 2017.

**Figure 1:**
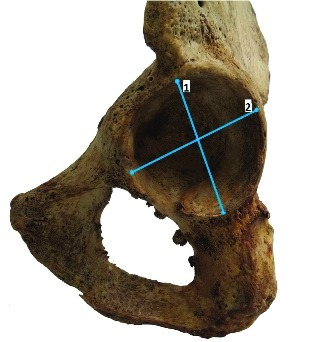
Measured parameters of the acetabulum: acetabular diameter – 1 and transverse acetabular diameter – 2.

By using a sliding caliper, the first author measured two acetabular parameters (Figure 1):

1) acetabular diameter (AD): maximum diameter of the acetabulum measured in a superior to inferior direction – diameter of the acetabulum along the axis of the body of the ischium (16).

2) transverse acetabular diameter (TAD): maximum acetabular diameter from the pubic eminence on the acetabular rim (17).

Both dimensions were measured to the nearest millimeter.

Normality of distribution was tested using Kolmogorov-Smirnov test. Both AD and TAD variables showed normal distribution on the left and right side in both sexes. The independent *t* test was used to compare measurements between men and women, and between the right and left acetabula of different individuals. After the original data set was collected, we determined the repeatability of measurement by the inter-observer test. The second author selected 40 acetabula from the original data set using True Random Number Generator (*Random.org*) and re-measured them. The two sets of values for these 40 individuals were compared with Pearson correlation coefficient r and dependent *t* test. Sexual dimorphism data are presented using standard descriptive statistics. Receiver operating characteristic (ROC) curve analysis was performed to determine the cut-off value for male sex prediction. Discriminant function analysis was performed by using both measured variables. The data were analyzed with SPSS, version 25.0 (IBM Corp, Armonk, NY, USA).

## Results

There were no significant differences between the left and right acetabula in both sexes (independent *t* test, *P* = 0.157). Both measured variables showed significant sexual dimorphism. AD and TAD values were significantly higher in men than in women (*P* < 0.001). Men exhibited greater variation than women for TAD, but not for AD. These results suggest that the measured variables are useful in evaluating morphological differences between the sexes (Table 1).

**Table 1:** Descriptive statistical analysis of acetabular diameter and transverse acetabular diameter for both sexes

		Arithmetic mean	Standard deviation	Minimum	Maximum	25th centile	Median	75th centile	t	Degree of freedom	*P*
Acetabular diameter	female	51.95	2.59	47.00	59.00	50.00	52.00	54.00	-14.2	198	<0.001
	male	57.12	2.56	52.00	63.00	55.00	57.50	59.00			
Transverse acetabular diameter	female	50.72	2.05	46.00	56.00	50.00	51.00	52.00	-14.8	198	<0.001
	male	55.76	2.73	50.00	62.00	54.00	56.00	58.00			

The cut-off value for prediction of male sex for AD was higher than 54 mm, and for TAD was higher than 52 mm. Therefore, the highest accuracy for AD was observed at specificity of 90% and sensitivity of 84%, and for TAD at specificity and sensitivity of 88%. Since the sample consisted of an equal number of men and women, the overall accuracy of this method was 87% for AD and 88% for TAD (Table 2).

**Table 2:** Cut-off point values of acetabular diameter and transverse acetabular diameter in the prediction of male sex obtained by receiver-operating characteristic curve analysis

	Criterion	Sensitivity (%)	Specificity (%)	Area under the curve	*P*
Acetabular diameter	>54 mm	84.00	90.00	0.92	<0.001
Transverse acetabular diameter	>52 mm	88.00	88.00	0.93	<0.001

**Figure 2:**
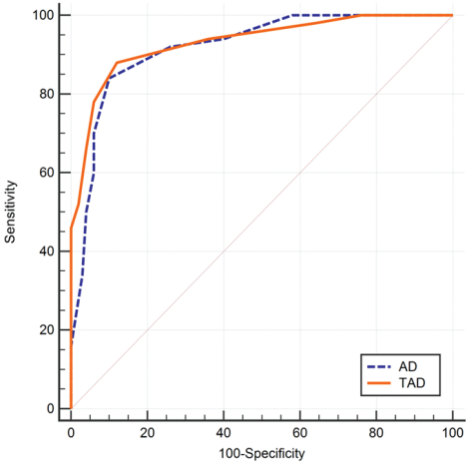
Receiver-operating characteristic curve for acetabular diameter and transverse acetabular diameter.

The area under the curve was large for both parameters and almost equal. This means that both parameters predicted male sex with similar accuracy (Figure 2).

The standardized coefficients indicate the relative contribution of each variable to the function. We used non-standardized coefficients to calculate discriminant function score from the raw data (Table 3). A discriminant score was obtained by multiplying each variable with its non-standardized coefficient, and adding them together along with the constant. The constant has no inherent value, and only serves to calibrate the sectioning point to zero when the number of men and women is equal. The discriminant function formula employing AD and TAD was the following:

**Table 3:** Standardized and non-standardized discriminant function coefficients, structure matrix, and classification for acetabular diameter and transverse acetabular diameter

Model	Structure matrix	Standardized canonical discriminant function coefficients	Non-standardized canonical discriminant function coefficients	Wilks' Lambda	*P*	Average accuracy
Acetabular diameter	0.967	0.431	0.168	0.495	<0.001	88% correctly classified (88% cross-validated)
Transverse acetabular diameter	0.932	0.618	0.256	0.476	<0.001	
Constant			-22.778			
Group centroids for females			-1.079			
Group centroids for males			1.079			
Sectioning point			0.000			

Df = -22.8 + 0.17 × AD + 0.26 × TAD

All values higher than 0.00 indicate male sex, while all values lower than 0.00 indicate female sex. The accuracy of sex estimation using this discriminant function was 88%.

Inter-observer error for measurements performed by two different authors was not significant (*P* = 0.858 for AD, *P* = 0.126 for TAD). There was a strong correlation between measurements obtained by two observers for both AD (r = 0.963) and TAD (r = 0.937). This indicates that both measurements were repeatable and could be measured with a high degree of accuracy.

## Discussion

The 87% accuracy of sex prediction with AD and 88% with TAD obtained in our study show that the acetabulum is a very good indicator for sex estimation in contemporary Croatian population. The cut-off points for AD correctly classified 90% of women (“specificity”) and 84% of men (“sensitivity”), while the cut-off points for TAD correctly classified 88% of women (“specificity”) and 88% of men (“sensitivity”). Employing both variables in sex discrimination function formulae leads to the accuracy of sex estimation in 88% of cases. Hence, with these two simple-to-use variables, we achieved a high level of accuracy necessary for working with skeletal remains in forensic context (6) and court procedures (18,19). Interestingly, the discriminant function formula using both AD and TAD can achieve hardly better accuracy than AD and TAD separately. This implies that sexual dimorphism in our specimen is almost entirely size-based, with only a minor influence of the shape of the acetabula.

While conducting the forensic analysis of a high number of skeletal remains from the Croatian War of Independence (over 4000 examined skeletal remains), we have often found the acetabulum to be well preserved, even after a long postmortem period. We measured either the left or right acetabulum of one individual and obtained an almost equal number of measurements for both sides. This is why we believe that the results can be used on both sides, especially because no significant difference was found between the groups of the left and right acetabula.

Our results are in accordance with the previous research in contemporary Croatian population, which showed that the maximum femoral head diameter (complementary part of the hip) can be used to discriminate the sex with 94% accuracy (20).

Acetabulum sex dimorphism has been studied in various populations. Our results can be compared to those obtained by Goméz-Valdéz et al (21), who have shown that TAD was one of the important sex indicators in the contemporary Mexican population, with the accuracy of almost 87%. A slightly higher accuracy obtained when using AD in Croatian population can suggest some sort of population specificity. Murphy (22) analyzed a sample of Polynesian innominate bones, using a single variable – maximum diameter of the acetabulum, which yielded 86% accuracy of sex determination. Stayn and Iscan (23) have established that the use of maximum acetabulum diameter, in a superior-inferior direction, in modern Greek population achieves the accuracy of 84%. Benazzi et al (11) achieved the accuracy of 96.4% based on the planar image of the acetabulum and related metric data measuring area, perimeter, longitudinal, and transverse maximum width.

The mentioned research indicates inter-population variability. We believe that the standards elaborated in this study are not suitable for use in forensic analysis of skeletal remains of populations other than Croatian. If our measurements are compared to the those obtained by Goméz-Valdéz et al (21) (mean AD and TAD for Croatian vs Mexican population for women are 51.9 vs 50.7 and 48.8 vs 46.3 respectively; and for men 57.1 vs 54.5 and 55.7 vs 52.2 mm respectively), the need for population specific results becomes obvious – Croatians of both sexes have on average larger AD and TAD than Mexicans. We believe, however, that our results could be applied to similar populations; primarily to other contemporary populations in Southeastern Europe. We also believe that these results can be used in other populations with a similar or the same sample mean and demarking points.

In conclusion, our results clearly show that acetabular measurements can be used for sex estimation in contemporary Croatian population. They are particularly important in cases of fragmented and incomplete skeletal remains when the acetabular region is preserved. The need for this is obvious since in Croatia there are still over 1500 missing and unidentified war victims (24). These findings will also be of special interest in routine medicolegal investigations. The results from this study encourage the development of new osteometric standards for sex determination based on other parts of the skeleton in contemporary Croats.

## References

[1] Ubelaker DH. Methodological considerations in the forensic applications of human skeletal biology. In: Katzenberg MA, Saunders SR, editors. Biological anthropology of the human skeleton. New York: Wiley-Liss; 2000. p. 41-67.

[2] Ubelaker DH, Volk C (2002). A test of the Phenice method for the estimation of sex.. J Forensic Sci.

[3] Rösing FW, Graw M, Marré B, Ritz-Timme S, Rothschild MA, Rötzscher K (2007). Recommendations for the forensic diagnosis of sex and age from the skeletons.. Homo.

[4] Mishra SR, Singh PJ, Agrawal AK, Gupta RN (2003). Identification of sex of sacrum of Agra region.. J Anat Soc India.

[5] St. Hoyme LE, Iscan MY. Determination of sex and race: accuracy and assumptions. In: Iscan MY, Kennedy KAR, editors. Reconstruction of life from the skeleton. New York: Alan R. Liss Inc; 1989. p. 53-93.

[6] Scheuer L (2002). Application osteology to forensic medicine.. Clin Anat.

[7] Cattaneo C (2007). Forensic anthropology: developments of a classical discipline in the new millennium.. Forensic Sci Int.

[8] Charisi D, Eliopoulos C, Vanna V, Koilias CG, Manolis SK (2011). Sexual dimorphism of the arm bones in a modern Greek population.. J Forensic Sci.

[9] Bidmos MA, Dayal MR (2003). Sex Determination From the Talus of South African Whites by Discriminant Function Analysis.. Am J Forensic Med Pathol.

[10] Iscan MY (1988). Rise of forensic anthropology.. Am J Phys Anthropol.

[11] Benazzi S, Maestri C, Parisini S, Vecchi F, Gruppioni G (2008). Sex assessment from the acetabular rim by means of image analysis.. Forensic Sci Int.

[12] Steyn M, Pretorius E, Hutten L (2004). Geometric morphometric analysis of the greater sciatic notch in South Africans.. Homo.

[13] Ari I (2005). Morphometry of the greater sciatic notch on remains of male Byzantine skeletons from Nicea.. Eur J Anat.

[14] Steyn M, Patriquin ML (2009). Osteometric sex determination from the pelvis – Does population specificity matter?. Forensic Sci Int.

[15] Macaluso PJ (2010). Sex determination from the acetabulum: test of a possible non-population-specific discriminant function equation.. J Forensic Leg Med.

[16] Kelley MA (1979). Sex determination with fragmented skeletal remains.. J Forensic Sci.

[17] Arsuaga J, Carretero J (1994). Multivariate analysis of the sexual dimorphism of the hip bone in a modern human population and in early hominids.. Am J Phys Anthropol.

[18] Rogers TL (2005). Determining the sex of human remains through cranial morphology.. J Forensic Sci.

[19] Williams BA, Rogers TL (2006). Evaluating the accuracy and precision of cranial morphological traits for sex determination.. J Forensic Sci.

[20] Slaus M, Strinović D, Skavić J, Petrovecki V (2003). Discriminant function sexing of fragmentary and complete femora: standards for contemporary Croatia.. J Forensic Sci.

[21] Gómez-Valdés JA, Torres Ramírez G, Báez Molgado S, Herrera Sain-Leu P, Castrejón Caballero JL, Sánchez-Mejorada G (2011). Discriminant function analysis for sex assessment in pelvic girdle bones: sample from the contemporary Mexican population.. J Forensic Sci.

[22] Murphy AM (2000). The acetabulum: sex assessment of prehistoric New Zealand Polynesian innominates.. Forensic Sci Int.

[23] Steyn M, Işcan MY (2008). Metric sex determination from the pelvis in modern Greeks.. Forensic Sci Int.

[24] Missing persons in the Croatian war for independence [in Croatian]. Available from: https://branitelji.gov.hr/o-ministarstvu/djelokrug/mjere/nestale-osobe/nestale-osobe-u-domovinskom-ratu-834/834*.* Accessed: March 22, 2019.

